# Disentangling photoperiod from hop vernalization and dormancy for global production and speed breeding

**DOI:** 10.1038/s41598-019-52548-0

**Published:** 2019-11-05

**Authors:** William L. Bauerle

**Affiliations:** Department of Horticulture and Landscape Architecture, Graduate Degree Program in Ecology, Colorado State University, Fort Collins, CO 80523 USA

**Keywords:** Agroecology, Ecophysiology

## Abstract

*Humulus lupulus* L. (hop) flowers are a key ingredient in beer, imparting the beverage’s aroma and bitterness profile. Photoperiod is known to interact with temperature to control flowering in hops. Studies have stipulated that resting dormant buds on hops require a minimum chilling duration for their meristems to break dormancy and grow fruitfully. This assertion, in part, led to a long-held notion that hops require vernalization and/or dormancy for the meristem to change from a vegetative to floral state. The research in this study aims to separate photoperiod from vernalization and dormancy through a series of experiments that artificially control photoperiod to prevent the onset of dormancy and chilling exposure. Six experiments were performed to assess flower yield and quality for seven diverse hop cultivars (with and without exposure to chilling and dormancy) to quantify the impact on flowering performance. Vernalization and dormancy, two plant traits previously considered necessary to the proliferation of hop flowers, do not influence hop flower yield and quality. The findings have broad implications; global hop production can be distributed more widely and it paves the way for speed breeding and controlled-environment production to achieve 4 hop generation cycles per year, as opposed to 1 under field-grown conditions.

## Introduction

Temperature and photoperiod are key climate constraints that limit hop (*Humulus lupulus* L.) flower production to within the 35–55 latitude^1–3^. Photoperiod, the first hop climate constraint to be recognized, was identified by Tournois (1912). In the century since, numerous studies emphasize the influence of temperature on hop quality and yield^2,3^. Photoperiod and temperature constrain hop production to just a few select microclimates (e.g. Yakima Valley, USA; Zatec, Czech Republic). Given that the yield and quality of the global hop supply are heavily influenced by these two climate factors, modern controlled environment technologies could be adopted to modify microclimate conditions and offer a broadly applicable hop production alternative.

In native conditions, hops are perennials that annually produce inflorescences, commonly called cones, approximately over a four month season. Changes in day length, which are at the greatest around the summer solstice, provide the environmental signal for hops to transition from the vegetative to regenerative growth stage^1,4,5^. Similarly, increases in the length of darkness are crucial for the qualitative phase transition in hop; flower primordia initiate only when photoperiods are shorter than a critical day length^4,5^.

Photoperiod is known to interact with temperature to control flowering in hops. In the field, the start of spring with warmer temperatures and longer photoperiods bring the hop out of dormancy. As temperature increases through the summer, the plant transitions from a juvenile to adult state. Warmer temperatures then permit hops to flower under shorter day lengths^4^. Ultimately, the increasingly shorter autumn day lengths initiate a gradual shoot death and the onset of dormancy by early winter^6^. At this point, vernalization completes the cycle, the process by which plants become competent to flower after exposure to prolonged winter chilling e.g.^7,8^.

In one of the seminal books on hops, Neve and colleagues stipulated that resting dormant buds on hops require a minimum chilling duration of 42 days at 3 °C for their meristems to break dormancy and grow fruitfully^9^. From their reports on chilling and dormancy, this assertion, in part, led to a long-held notion that hops require vernalization and/or dormancy for the meristem to change from a vegetative to floral state. Although vernalization and dormancy have been accepted as flower induction prerequisites for hops e.g.^9,10^, the author knows of no published data to substantiate or refute the necessity for either low temperature chilling or a dormant phase and no published data are available that equate chilling hours with hop flower induction and proliferation.

The challenges of growing hops in a controlled environment under confined conditions might be partly responsible for the lack of information on hop chilling and dormancy requirements. For most species requiring vernalization, there is a process of resetting to the default state, such that plants of the next crop cycle will not flower unless the perennial organ is exposed to a chilling period^7,8,11^. Hops, being annual climbing bines and perennial rootstocks, return to the juvenile phase per generation^9,12^. In comparison with small stature perennial species that require vernalization, hop bines have a protracted juvenile phase during which they are incapable of flowering unless 12–25 nodes are visible to the naked eye^4^. This is due to hops requiring a variety-specific size effect (distance) between the roots and shoot apex to make the juvenile-adult transformation^4^. This stipulation results in bines that are of significant length and morphological complexity, making them difficult subjects for experimental work in a confined space.

The research in this study separates photoperiod from vernalization and dormancy through a series of experiments that artificially control photoperiod to prevent the onset of dormancy and chilling exposure. We hypothesized that photoperiod is the sole environmental trigger for the proliferation of hop flowers and that vernalization and dormancy are not necessary in the growth of hop flowers, two factors that currently restrict hop global production to just a few select microclimates. Six experiments were performed to assess flower yield and quality for seven diverse hop cultivars (with and without exposure to chilling and dormancy) to quantify the impact on flowering performance. There were no statistically significant effects of vernalization on the cone yield among cultivars in rootstock, cutting, and tissue culture initiated bines. By increasing the photoperiod above the critical length for hop flower induction in both vernalized and non-vernalized plant material, we show that hops do not require either low temperature chilling or dormancy to achieve typical flower initiation, formation, and cone yield. Thus, there has been a hop breeding and production setback due to the fallacy of a vernalization and dormancy requirement.

## Results and Discussion

**Figure 1:**
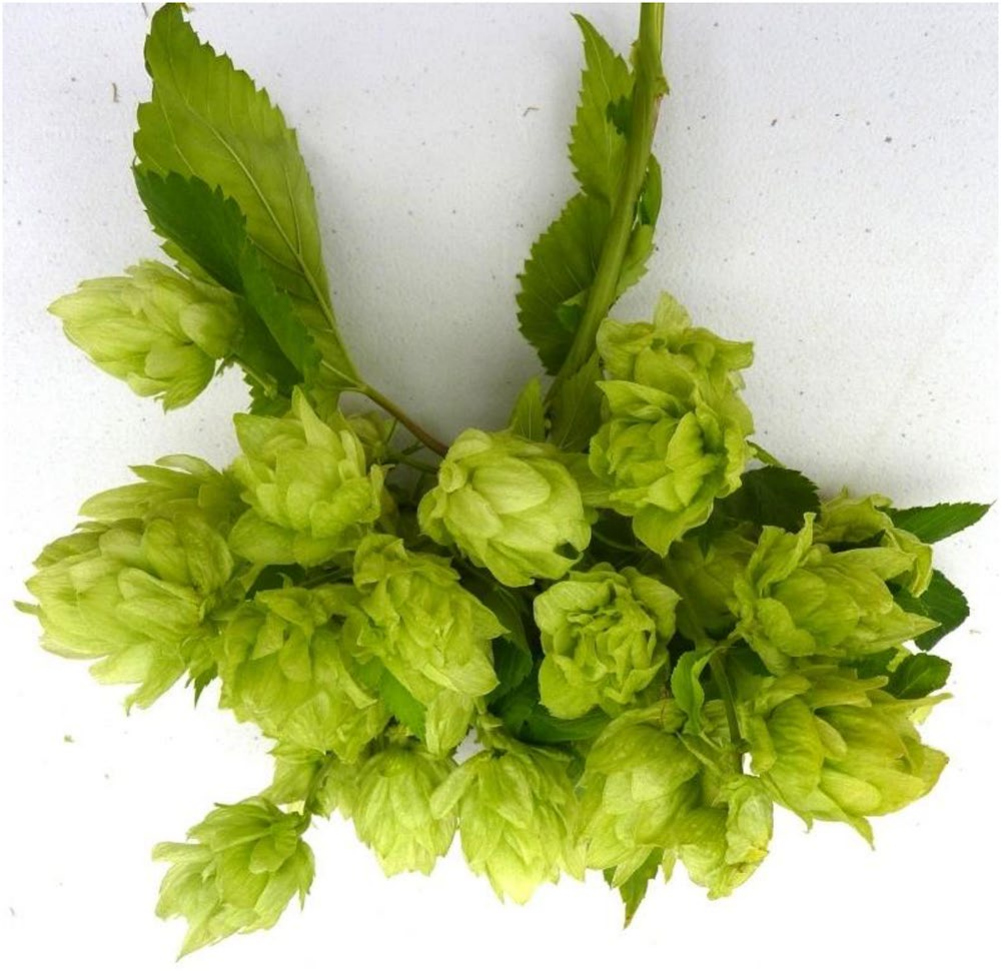
Hop cone abundance at the terminal node of a lateral. Numerous cones developing on a non-vernalized 67 day old tissue culture generated cv. ‘Centennial’ bine.

**Figure 2:**
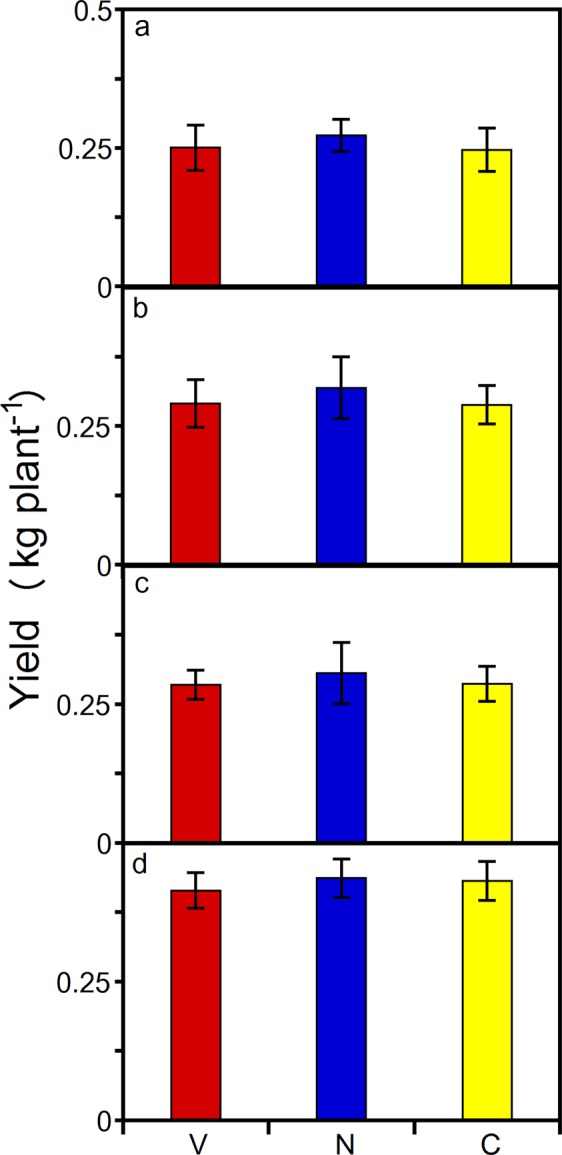
The hop yield with natural vernalization and dormancy versus without. Vernalized rootstock (V), non-vernalized rootstock (N), and non-vernalized softwood cutting (C) dry cone yield (kg plant^−1^). (**a**) cv. ‘Cascade’. (**b**) cv. ‘Centennial’. (**c**) cv. ‘Chinook’. (**d**) cv. ‘Columbus’. Vertical bars represent standard deviations of seven replicates (n = 7). Means are not statistically different from each other at *P* < 0.05 (one-way ANOVA).

**Figure 3:**
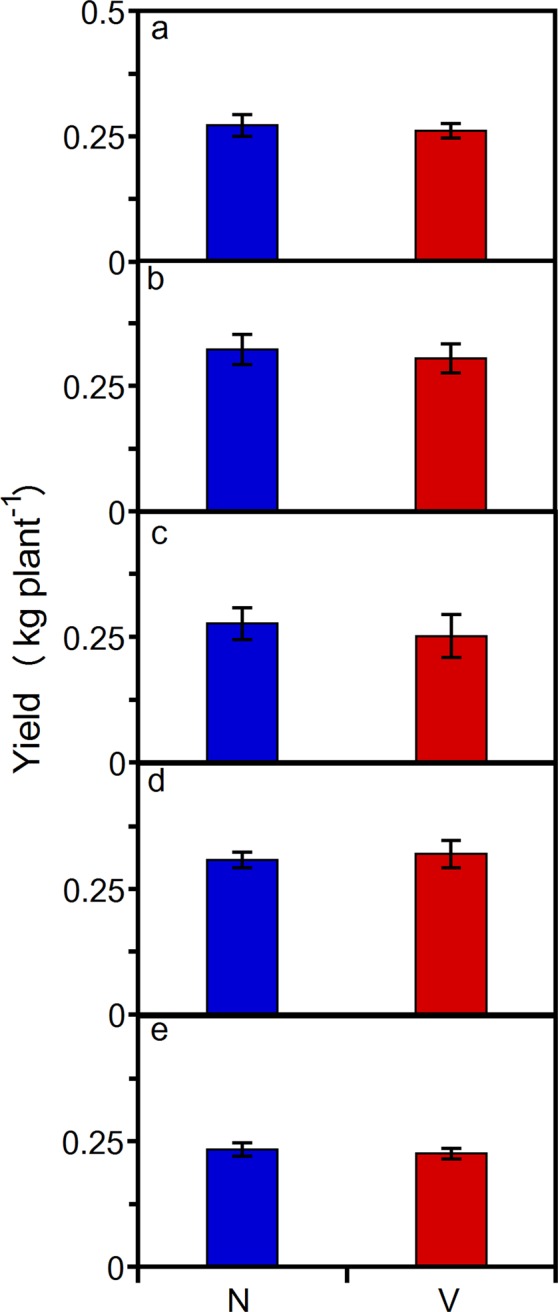
The hop yield with controlled vernalization and dormancy versus without. Non-vernalized (N) and vernalized (V) tissue culture generated plantlet dry cone yield (kg plant^−1^). (**a**) cv. ‘Cascade’. (**b**) cv. ‘Centennial’. (**c**) cv. ‘Chinook’. (**d**) cv. ‘Galena’. (**e**) cv. ‘Willamette’. Vertical bars represent standard deviations of seven replicates (n = 7). Means are not statistically different from each other at *P* < 0.05 (one-tailed unequal variances t-test).

Visually, it was apparent that the yields did not suffer. For example, the cone amount at the terminal node of a non-vernalized cv. ‘Centennial’ lateral and the cone quantity on two lateral side-shoots at a representative main bine node of a non-vernalized cv. ‘Willamette’ bine were visually high-yielding (Fig. 1 and Supplementary Image Fig. S1). In addition, a subsection of cv. ‘Centennial’ canopy with cones in the ripening stage and an individual portion of a lateral side shoot further illustrate the quantity of cones produced on non-vernalized bines (Supplementary Image Figs S2 and S3). The vernalized and non-vernalized rootstock cone yield for cv. ‘Cascade’, ‘Centennial’, ‘Chinook’, and ‘Columbus’ were not different (Fig. 2a–d; one-way ANOVA’s (a) *P* = 0.38; (b) *P* = 0.39; (c) *P* = 0.56; (d) *P* = 0.45, respectively). More importantly, under controlled-environment conditions non-vernalized softwood cutting propagules achieve similar yields to vernalized or non-vernalized rootstock-generated bines at approximately four months of age (Fig. 2a–d; unequal variance paired t-test’s (a) *P* = 0.42 and *P* = 0.1; (b) *P* = 0.45 and *P* = 0.12; (c) *P* = 0.46 and *P* = 0.22; (d) *P* = 0.18 and *P* = 0.4, respectively). Comparably, Fig. 3a–e illustrates non-vernalized tissue culture plantlets compared to vernalization in the following crop cycle. There were no statistically significant effects of vernalization on the cone yield for cv. ‘Cascade’, ‘Centennial’, ‘Chinook’, ‘Galena’, and ‘Willamette’ (unequal variance paired t-test’s a) *P* = 0.16; b) *P* = 0.14; c) *P* = 0.13; d) *P* = 0.17; e) *P* = 0.12, respectively). Per crop cycle, the controlled-environment cone-yield values were 23.7–72.9% higher per unit land area than those reported for commercial field production and the variety specific cone yield follows the same yield gradient as that observed under field conditions (Table 1). Lastly, the cone yield was within the range observed in the same and comparable USDA selections (e.g. ‘Galena’)^13^.

**Table 1:** Field-grown versus observed controlled-environment grown dry cone yield of examined hop cultivars.

Cultivar	Field yield kg ha^−1^	Observed yield kg ha^−1^	% difference of field
*‘Cascade’*	2,210	2,805 ± 57	+23.7
*‘Cashmere’*	1,703	3,633 ± 76	+72.3
*‘Centennial’*	1,529	3,284 ± 75	+72.9
*‘Chinook’*	2,102	3,024 ± 95	+35.9
*‘Columbus’*	2,782	4,597 ± 72	+49.2
*‘Galena’*	2,409	3,379 ± 63	+33.5
*‘Willamette’*	1,511	2,468 ± 44	+48.1

Field grown yield^24^, observed yield (this study), and percentage difference (%) of the highest field cone yield (Washington State, USA 2018)^24^ versus controlled-environment observed yield (this study). Controlled-environment observed yield occurred at a bine density of 0.93 m^2^ per ha^−1^. Field bine density typically ranges between 5.0–2.5 m^2^ per ha^−1^ (estimated at 2–4 bines per hill at 2,500 hills per ha^−1^). Cone yield and standard error terms among crop cycles were pooled per cultivar and standard errors reported as the difference of the means for cv. ‘Cascade’ (n = 35, experiment 1–5), ‘Cashmere’ (n = 7, experiment 6), ‘Centennial’ (n = 35, experiment 1–5), ‘Chinook’ (n = 35, experiment 1–5), ‘Columbus‘ (n = 21, experiment 1–3), ‘Galena’ (n = 14, experiment 4–5), and ‘Willamette’ (n = 14, experiment 4–5). Note, observed versus field-grown yield comparisons represent one crop cycle per annum.

**Figure 4:**
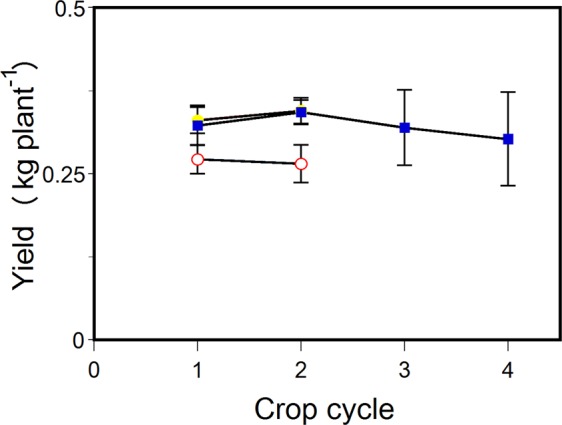
Dried hop cone yield (kg plant^−1^) of successive crop cycles of non-vernalized tissue culture generated plantlets. Cv. ‘Cascade’ (open circle; n = 7), ‘Cashmere’ (closed circle; n = 7), and ‘Centennial’ (closed square; n = 7). Vertical bars represent standard deviations. Means are not statistically different from each other at p = 0.05 for ‘Cascade’ and ‘Cashmere’ (t-test) and ‘Centennial’ (one-way ANOVA).

A key concern for container experiments is providing a large enough rooting volume to minimize ‘pot effects’ across crop cycles. Insufficient rooting volumes can result in water stress even when plants are ‘well-watered’ if atmospheric demands are high. The “peril of pot experiments” was highlighted by Passioura^14^. We attempted to eliminate this artifact as much as possible by using ample pot volumes of similarly sized glasshouse grown crops (e.g. cucumber, tomato). Nonetheless, we tested the potential effect of container constraints on bine growth and cone yield by repeating rootstock crop cycles in succession. The cultivar specific bine growth rate and total visible node development of the controlled-environment grown plants (with and without exposure to chilling and/or dormancy) are illustrated in supplementary data (Fig. S4). Bine length and total visible node development in the controlled-environment grown cultivars are similar to and less variable than values among five annual cycles reported for field-grown hops^15^. Under regulated environmental conditions, we also observed a relatively consistent, albeit cultivar specific, growth rate and node development progression among crop cycles - regardless of the presence or absence of vernalization and dormancy (Supplementary Data Fig. S4). In a companion study, non-vernalized tissue-culture plantlets were used to test for a reduction in yield between successive 90 day crop cycles (Fig. 4). We did not find a significant difference in inflorescence yield between crop cycle 1 and 2 (‘Cascade’ *P* = 0.32, ‘Cashmere’ *P* = 0.1, and ‘Centennial’ *P* = 0.08; paired t-test) or across four successive crop cycles in cv. ‘Centennial’ (*P* = 0.49; one-way ANOVA) (Fig. 4).

Ultimately, the brewing characteristics of field-grown hops are the benchmark for comparison to those grown under controlled-environment conditions. The analysis of α and β acids present in hop flowers is an important tool in elucidating the chemical quality of hop cones because brewers use the acid concentrations to determine cone quality. Additionally, the levels of α and β acids in the hop inflorescence are used to calculate the bitterness profile in the end product - beer. Comparing the typical range of cultivar specific α and β-acid values to the inflorescence chemical characteristics observed showed that α and β acids were not adversely impacted by controlled-environment conditions (Table 2). Furthermore, the comparison among cultivar-specific values cited in the literature with the values in this study indicate that the concentrations of α and β acids grown under controlled-environment conditions were within the typical cultivars’ α and β-acid range (Table 2)^16–21^. It is noteworthy that little variation in cultivar-specific cone chemical constituents and yield occurred among controlled environment experiments. “Climate variation is widely known” to effect field-grown hop flavor and bitterness^22,23^ as well as yield and quality^2,3,23–25^, whereas controlled-environment conditions moderate environmental fluctuation to minimize the variation in hop yield and quality among crop cycles.

**Table 2:** Typical versus observed alpha and beta-bitter acids of examined hop cultivars.

Cultivar	α acid typical	α acids observed	β acid typical	β acids observed
*‘Cascade’*	4.4–6.5	7.1 ± 0.6	4.5–6.5	6.5 ± 1.1
*‘Cashmere’*	7.7–9.1	7.9 ± 0.4	6.4–7.1	6.4 ± 0.9
*‘Centennial’*	9.5–11.5	9.6 ± 0.8	3.5–4.5	3.2 ± 0.4
*‘Chinook’*	12.0–14.0	14.3 ± 0.5	3.0–4.0	4.1 ± 0.3
*‘Columbus’*	14.5–16.5	18.9 ± 1.2	4.0–5.0	3.7 ± 0.2
*‘Galena’*	11.5–13.5	10.5 ± 1.7	7.2–8.4	7.6 ± 0.6
*‘Willamette’*	4.0–6.0	4.7 ± 0.5	3.5–4.5	3.8 ± 0.3

Field-grown (ranges)^13,16–21^ versus non-vernalized observed means of n = four 100 g cone subsamples from each cultivar ± standard deviation. Acid values are cone dry weight percentages.

The global beer supply is vulnerable to climate change^26^. We show that hop yield and quality were not significantly different with chilling and dormancy exposure as opposed to the lack of vernalization and dormancy. This provides evidence that two flowering cues, dormancy and vernalization, are not necessary in the growth of hop flowers. Additionally, in a controlled environment, it was possible to produce 4 uninterrupted crop cycles per annum from the same rootstock (e.g. Fig. 4). The findings unveil the possibility of distributing global hop production more widely, improving hop yield and quality attributes via speed breeding selections throughout the year^26^, and increasing production to alleviate risk in the supply chain.

## Materials and Methods

### Environmental conditions

Primary measurements occurred during 2016–2018 in the Horticulture Center at Colorado State University, Fort Collins, CO where a combination of environmentally controlled refrigerators and greenhouses allowed for controlled studies under defined conditions. Greenhouse conditions were programmed to a set point air temperature of 26 °C during photoperiod and 20 °C during the dark with a 45 minute temperature step change between the two, 50% relative humidity (RH; %), and supplemental photosynthetically active radiation (PAR) of 100–200 umol m^−2^ s^−1^ during the photoperiod (Philips LED lighting, Amsterdam, The Netherlands). Controllers were programmed to permit the maximum amount of light penetration (shade cloth was only pulled when intense solar radiation and temperatures demanded additional cooling efforts) where daytime PAR was generally 800–1,100 µmol m^−2^ s^−1^. Daytime temperatures over the experimental period averaged 26.4 °C, but in some instances temperatures climbed higher despite continuous cooling. Supplemental humidity was provided via an evaporative cooling pad, and the daytime saturation vapour pressure deficit (VPD) averaged 1.4 kPa. Air temperature and RH were measured using EHT RH/temperature sensors and PAR using AQO-S PAR photon flux sensors mounted at the top of the canopy (Decagon Devices, Pullman, WA, USA).

### Plant material

Over the course of the study, female varieties of seven hop cultivars were used. The varieties were selected from public cultivars that represent both aroma and alpha varieties. Their origin, indicative harvest time, and brewing use are reported in Supplementary Data Table S1. In a pilot experiment, the minimum number of nodes for flower induction - to not confound juvenility with flowering potential and node number among crop cycles - was quantified along a gradient in plant sizes and visible node development. In so doing, plants of progressively greater node development were quantified from the base of the plant to the last visible node prior to the apical meristem. It was determined that all cultivars in the study are ‘ripe to flower’ when ≥25 nodes are visible to the eye.

### Experiment 1: naturally vernalized rootstock

In 2016, winter-dormant naturally vernalized rootstocks of hop cultivars ‘Cascade’, ‘Centennial’, ‘Chinook’, and ‘Columbus’ were placed in a controlled environment greenhouse in 11 L bato buckets containing 100% horticulture grade perlite under supplemental PAR of 100–200 umol m^−2^ s^−1^ and an extended photoperiod to control day length at 18 h (Philips LED lighting, Amsterdam, The Netherlands). Containers were spaced 0.61 m (within rows) in 20 m rows per cultivar with 1.52 m between rows. Upon shoot emergence, shoots were thinned and one bine per container was trained to a string trellis at approximately 0.5 m of initial bine length. Thus, bines in this study received 0.93 m^2^ of space per bine to minimize bine-bine interactions within a container or between neighboring plants. We note that although there is not a ‘standard’ bine spacing per unit area for hops, the plant density and controlled environment yield calculations in this study equated to 10,764 bines per ha^−1^, which is a plant density similar to manual versus mechanized field hop production^12^. Periodic lengthening of the string in the vertical direction permitted the base of the plant to be lowered around the container in order to accumulate growth and node development. This technique permitted bine lengths in excess of 10 m. An automated irrigation system supplied ample nutrient and water conditions by feeding with a complete fertilizer (15-2-24, Aagrozz Inc., Wooster, OH, USA) via pressure compensating drip emitters (ML Irrigation Inc., Laurens, SC, USA). Initially, all pots were watered to saturation and permitted to drain for 18 h and thereafter container capacity was maintained daily. White plastic sheeting was cut and placed on the substrate surface to eliminate evaporation and bines were lowered on an as need basis until a minimum of 25 visible nodes developed. Biological crop protection was deployed to control aphids, spider mites, and thrips by means of *Aphidius colemani*, *Phytoseiulus persimilis*, and *Orius insidiosus*, respectively (Biobest Group NV, België). Once ‘ripe to flower’, photoperiod was reduced to 14 h for flower induction and black out blinds were used to prevent light pollution.

### Plant growth and cone yield

Seven plants (n = 7) of each cultivar were randomly selected and sampled weekly for repeated measurements of bine growth and visible node development. The seven plants that were repeatedly measured were treated as replicates in the analyses and the remaining plants per cultivar were randomly dispersed among the sampled plants to act as buffers. At harvest, bines were cut at the substrate level and manually picked. Cone fresh weights were measured on a per replicate basis and four 100 g cone subsamples were taken from each cultivar at harvest for dry matter content. The subsamples were weighed fresh and then immediately dried at 45 °C in forced air until reaching approximately 9% moisture. The samples were then reweighed, vacuum-sealed in clear plastic bags, and stored at 2 °C for determination of chemical constituents. Cone yield per bine replicate was calculated for each cultivar.

### Cone acid analysis

Contents of α and β-acids were determined by the spectrophotometric technique of the American Society of Brewing Chemists^27^. Cones of each dried sample were ground to a fine powder per cultivar and a homogenized sample was extracted from the lot of dried raw hops; 2.5 g of dried hop powder was weighed to the nearest mg and placed in a 100 mL beaker with 50.0 mL of methanol. The aliquot was stirred for 30 min at room temperature and the extract was then force filtered via centrifuge to remove particulate matter. A 50 μL aliquot of the filtrate was placed in a 25 mL volumetric flask and the flask was then filled with methanolic NaOH (0.5 mL of 6 M NaOH in 250 mL of methanol). An aliquot of this solution was placed in a 1 cm quartz cell and its absorbance values obtained for the wavelengths of 275, 325, and 355 nm against a blank of 50 μL of methanol in 25 mL of methanolic NaOH (Hach 6000 spectrophotometer; Loveland, CO, USA).

### Experiment 2: non-vernalized rootstock

After the crop cycle and cone harvest in experiment 1 was complete, the rootstocks of ‘Cascade’, ‘Centennial’, ‘Chinook’, and ‘Columbus’ were returned to 18 h photoperiod and allowed to sprout new shoots in the controlled environment. The new emerged shoots were either harvested as softwood cuttings (for Exp. 3) or thinned to one bine per container to proceed with the next crop cycle. Thus, the new annual shoots emerged from rootstocks that were not reset by exposure to either atmospheric chilling or a rhizospheric dormant phase. The growing set-point conditions, bine growth, and cone yield and acid analysis were the same as experiment 1.

### Experiment 3: non-vernalized softwood cutting propagules

Softwood cuttings of ‘Cascade’, ‘Centennial’, ‘Chinook’, and ‘Columbus’ were harvested as previously described. Cuttings, two nodes in length (the lower pair of leaves removed), were rooted in an aeroponic propagator (Ez-clone Inc. Sacramento, CA, USA) at a water pH of 6.0. Two weeks were adequate to develop root systems. Rooted cuttings were transplanted into bato buckets as previously described and immediately grown to maturity at a photoperiod of 18 h. In so doing, the rooted shoot did not enter a dormant phase nor was the new shoot and root system exposed to atmospheric or rhizosphere chilling. The softwood generated bine growing conditions, growth, and cone yield and acid analyses were as for experiment 1.

### Experiment 4: non-vernalized tissue culture plantlets

We grew ‘Cascade’, ‘Centennial’ ‘Chinook’, ‘Galena’, and ‘Willamette’, from tissue culture propagated plantlets (Summit Plant Labs Inc., Fort Collins, CO, USA). The cultured plantlets did not rescind from a dormant phase nor were they exposed to chilling. The growing conditions, growth, and cone yield and acid analysis for the tissue culture generated bines were as for experiment 1.

### Experiment 5: temperature controlled vernalization and dormancy

After one crop cycle, we artificially vernalized a subset of ‘Cascade’, ‘Centennial’, ‘Chinook’, ‘Galena’, and ‘Willamette’ under dark conditions in cold storage at 3 °C. After a minimum of 6 weeks of chilling and dormancy, the containers were removed from the cooler and placed in the greenhouse under 18 h photoperiod. Growing conditions, growth, and cone yield and acid analyses were as for experiment 1.

### Experiment 6: non-vernalized successive crop cycles

We grew cv. ‘Cascade’ and ‘Cashmere’ in double and cv. ‘Centennial’ in quadruple successive crop cycles. In so doing, rootstocks were not reset by exposure to either atmospheric chilling or a rhizospheric dormant phase from one crop cycle to the next. In each crop cycle, growing conditions, growth, and cone yield and acid analysis were as for experiment 1.

### Statistical analysis

Plant response data were analyzed using SPSS (IBM Analytics, www.ibm.com/analytics/, USA). One-way analysis of variance (ANOVA) (exp. 1–3, 6 - cv. Centennial) and t-test’s (exp. 4–5, 6 - cv. ‘Cascade’ and ‘Cashmere’) were used to analyze the significance of main effects. Differences between means were considered significant when the *P* value of the ANOVA *F* test or the t-value was <0.05. Each tracked plant was an experimental unit and treated as a replicate in experiments 1–6 (n = 7).

## Supplementary information

Supplementary Information

## Data Availability

The datasets generated during and/or analyzed during the current study are available from the corresponding author on reasonable request.
