# Supplementary material for: Disentangling photoperiod from hop vernalization and dormancy for global production and speed breeding
Source: Sci Rep. 2019 Nov 5;9:16003. doi: 10.1038/s41598-019-52548-0 (PMC6831652; doi:10.1038/s41598-019-52548-0)
Supplement: Supplementary file 1 — Supplementary Information [file 41598_2019_52548_MOESM1_ESM.pdf]

Supplementary information

Disentangling photoperiod from hop vernalization and dormancy for global production and speed breeding

William L. Bauerle[1*]

[1]Department of Horticulture and Landscape Architecture, Graduate Degree Program in Ecology, Colorado State University, Fort Collins, CO 80523

*Corresponding author: William L. Bauerle

Correspondence email: bauerle@colostate.edu

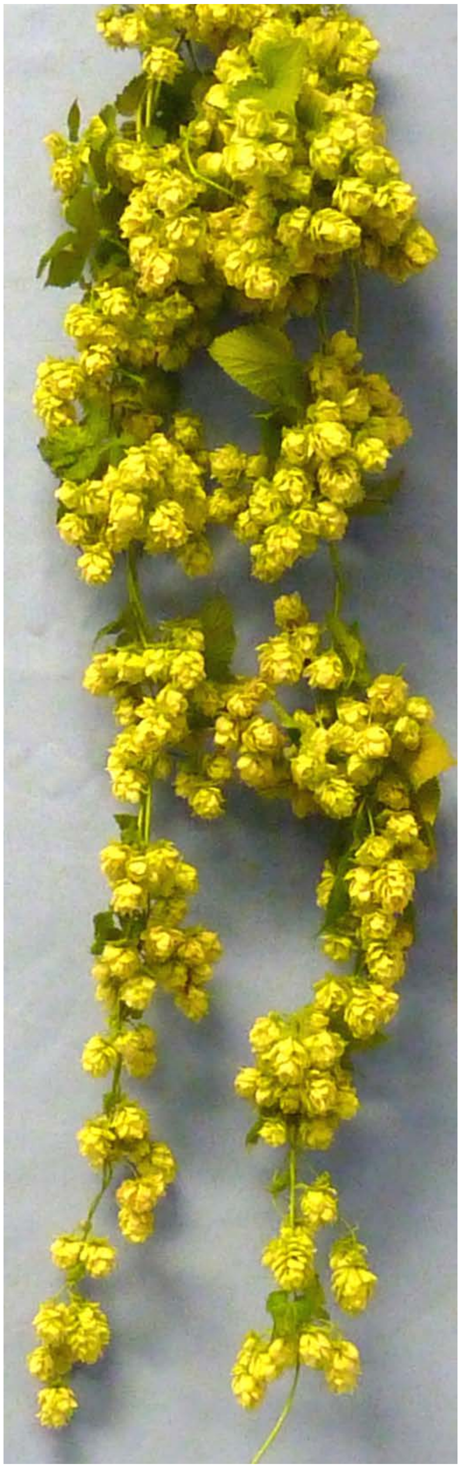

Supplementary Figure 1. Representative example of hop cone production at a node (two lateral side-shoots) of a non-vernalized three month old cv. ‘Willamette’ tissue culture generated bine. Note, flowers were ‘ripe’ at 90 days into the crop cycle.

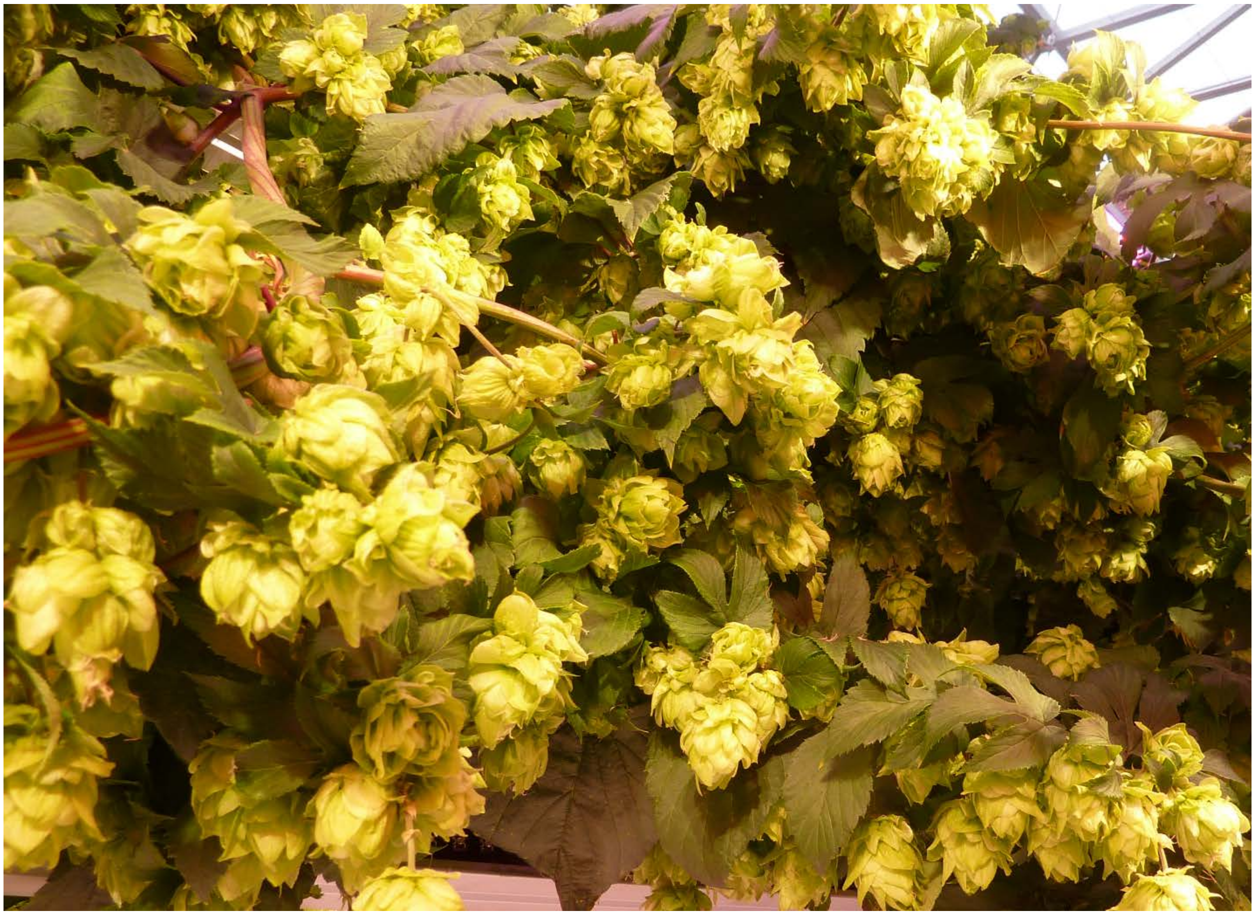

Supplementary Figure 2. Representative example of hop cone production on a non-vernalized cv. ‘Centennial’ crown subsection. Hop cone production at 67 days into the crop cycle.

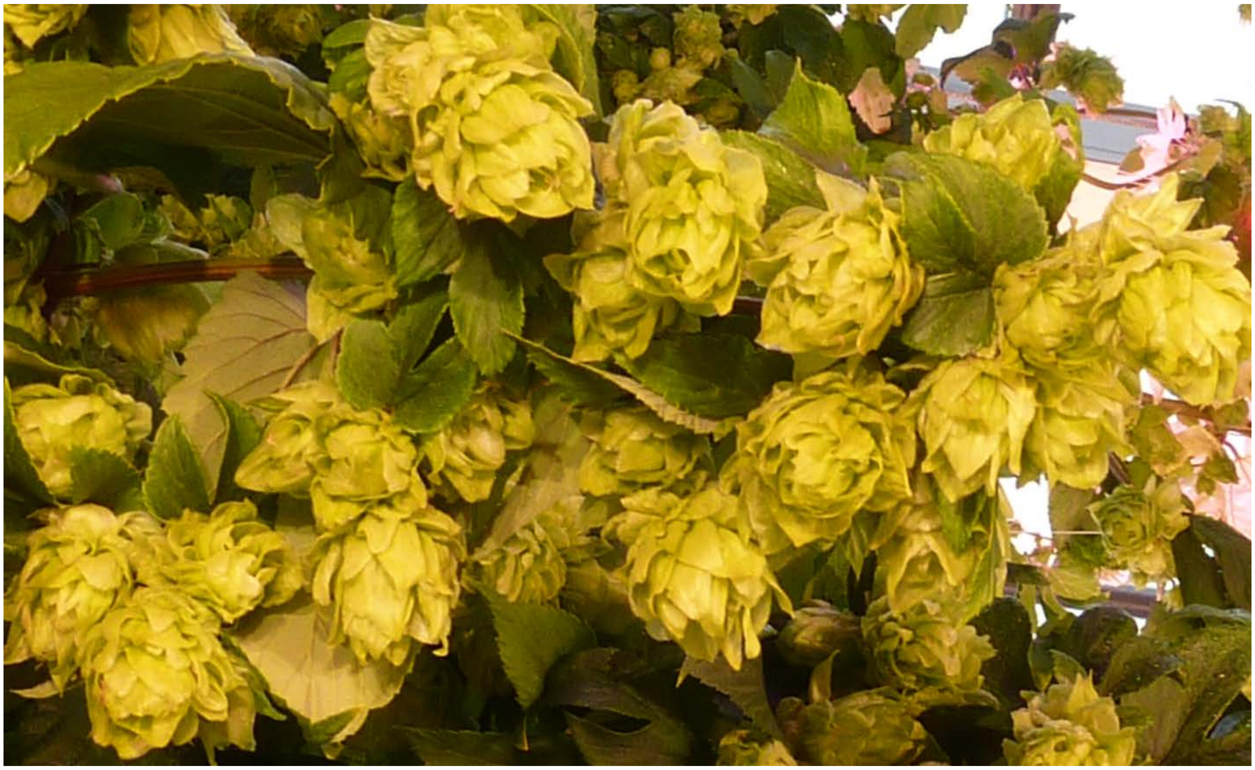

Supplementary Figure 3. Representative example of hop cone production at an individual portion of a lateral side shoot within a non-vernalized cv. ‘Centennial’ crown. Hop cone production after 67 days into the crop cycle.

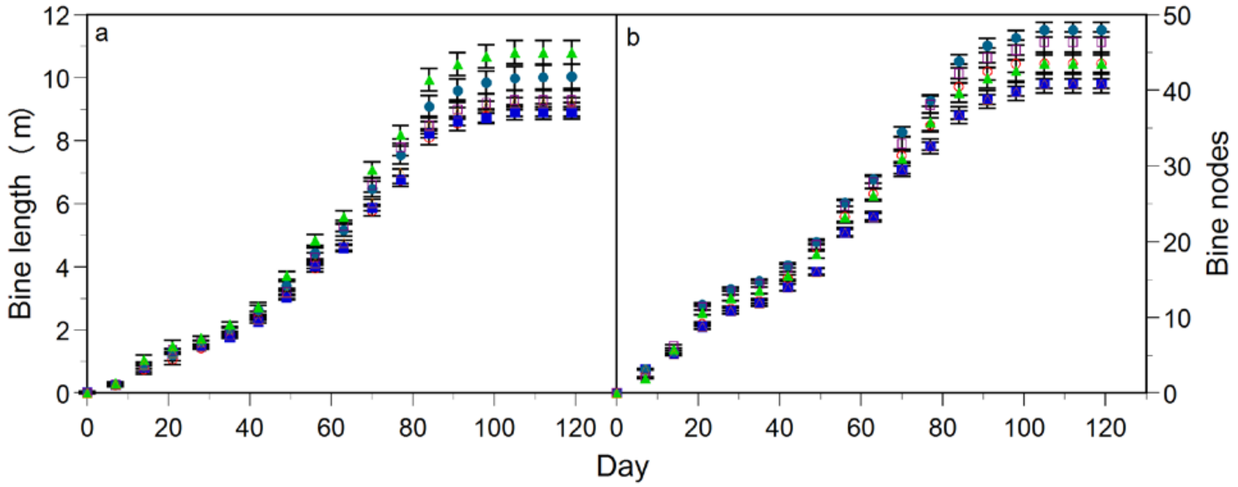

Supplementary Figure 4. *Humulus lupulus* L. a, bine length and b, total node number among controlled environment cycles. Cv. ‘Cascade’ (open circle), cv. ‘Centennial’ (closed square), cv. ‘Chinook’ (closed circle), cv. ‘Columbus‘ (open square), cv. ‘Galena’ (closed triangle), and cv. ‘Willamette’ (open triangle). Error terms among non-vernalized and vernalized crop cycles were pooled per cultivar and reported as standard error of the difference of the means for cv. ‘Cascade’ (n = 35), ‘Centennial’ (n = 35), ‘Chinook’ (n = 35), ‘Columbus‘ (n = 21), ‘Galena’ (n = 14), and ‘Willamette’ (n = 14). Vertical bars represent standard errors.

Supplementary Table 1. List of hop cultivars used in this study, their relative field-grown harvest time, brewing use, and year of public release. Harvest time: E= early, M = medium. NA = not available.

Cultivar	Harvest time	Brewing use	Year of release
‘*Cascade*’	M	Dual purpose	1972
‘*Cashmere*’	M	Aroma	2013
‘*Centennial*’	E - M	Dual purpose	1991
‘*Chinook*’	E - M	Dual purpose	1986
‘*Columbus*’	M	Bittering	NA
‘*Galena*’	E - M	Bittering	1979
‘*Willamette*’	E	Aroma	1977
